# North East London coronavirus disease 2019 protocol for diagnostics in two-week wait head and neck cancer patients

**DOI:** 10.1017/S0022215120001267

**Published:** 2020-06-18

**Authors:** E Warner, D W Scholfield, A Adams, P Richards, S Ali, J Ahmed, K Ghufoor

**Affiliations:** 1 Department of ENT, Royal London Hospital, Barts Health NHS Trust, UK; 2 Department of Radiology, Royal London Hospital, Barts Health NHS Trust, UK

**Keywords:** Head And Neck Neoplasms, Squamous Cell Carcinoma Of Head And Neck, COVID-19, Thyroid Neoplasms, Thyroid Nodule, Diagnostic Imaging, Histopathology

## Abstract

**Background:**

The coronavirus disease 2019 pandemic requires urgent modification to existing head and neck cancer diagnosis and management practices. A protocol was established that utilises risk stratification, early investigation prior to clinical review and a reduction in aerosol generating procedures to lessen the risk of coronavirus disease 2019 spread.

**Methods:**

Two-week wait referrals were stratified into low, intermediate and high risk. Low risk patients were referred back to primary care with advice; intermediate and high risk patients underwent investigation. Clinical encounters and aerosol generating procedures were minimised. A combined diagnostic and therapeutic surgical approach was undertaken where possible.

**Results:**

Forty-one patients were used to assess feasibility. Thirty-one per cent were low risk, 35 per cent were intermediate and 33 per cent were high risk. Thirty-three per cent were discharged with no imaging.

**Conclusion:**

Implementing this protocol reduces the future burden on tertiary services, by empowering primary care physicians to re-refer low risk patients. The protocol is applicable across the UK and avoids diagnostic delay.

## Introduction

The coronavirus disease 2019 (Covid-19) pandemic presents an unparalleled challenge to head and neck cancer services. Well-established protocols must be urgently modified to reduce the impact on head and neck cancer patient morbidity and survival. NHS England has responded to the Covid-19 pandemic by adapting existing cancer waiting time guidance to recommend telephone triage of referrals.^1^ The British Association of Head and Neck Oncologists recommends prioritising cases that are likely to represent malignancy and deferring cases with a lower likelihood.^2^

A risk calculator (the head and neck cancer risk calculator, version 2; ‘HaNC-RC v.2’) has been generated in order to establish the probability of head and neck cancer in individual patients.^3^ We have reviewed the head and neck cancer risk calculator, version 2, and feel that this is a valuable way of triaging patients.

The ENT UK two-week wait telephone triage service evaluation advises deferring low risk patients until after the pandemic or discharging them.^4^ However, there may be future resource implications of deferring patients, as the National Health Service is likely to struggle to reinstate all the services that have been postponed. Furthermore, working through the back-log of cases will take over a year, before normal service is resumed.^5^ Low risk patients should be empowered to seek referral back if symptoms have not settled, through the primary care route, rather than further virtual review at six months. Clearly, individual clinical judgement needs to be used alongside the calculator, to assess risk.

We have constructed a protocol to streamline investigations during the Covid-19 pandemic, limiting flexible nasendoscopy and diagnostic endoscopy to where absolutely necessary. Surgical management has been adapted to reduce the risk of transmission, minimising aerosol generating procedures. If possible, tissue diagnosis should take place in the clinic via intra-oral biopsy, transnasal oesophagoscopy aided biopsy, ultrasound-guided (percutaneous) fine needle aspiration (FNA) or core biopsy. If these methods are not successful in yielding tissue, a combined diagnostic and therapeutic surgical approach should be undertaken where appropriate, in order to reduce the number of surgical procedures required to definitively manage patients.

## Materials and methods

**Fig. 1.**
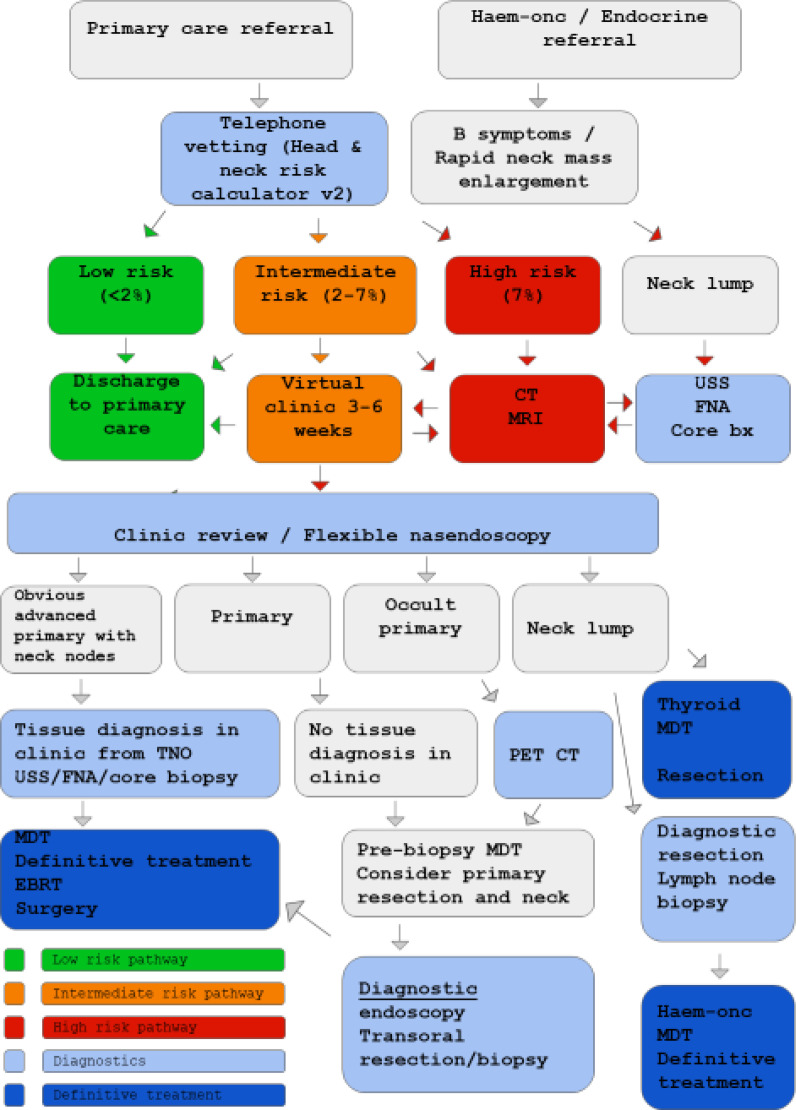
Flow chart of the North East London Covid-19 protocol for diagnostics in two-week wait head and neck cancer patients. Haem-onc = haematology-oncology; v2 = version 2; CT = computed tomography; MRI = magnetic resonance imaging; USS = ultrasound scan; FNA = fine needle aspiration; TNO = transnasal oesophagoscopy; PET-CT = positron emission tomography computed tomography; MDT = multidisciplinary team; EBRT = external beam radiotherapy

Risk stratification of all patients should take place after referral for suspected head and neck cancer (Figure 1). Symptoms and risk factors are elicited via telephone consultation, and used to complete the head and neck cancer risk calculator, version 2.^3^ If the patient is categorised as low risk (less than 2 per cent risk of cancer), they are discharged to primary care with safety-net advice. The patient should be empowered to seek review by their general practitioner in three months and re-refer if they remain symptomatic. For intermediate risk (2–7 per cent probability of cancer) patients, clinician judgement should be used to decide whether patients can be safely discharged to their general practitioner, or whether imaging is indicated prior to further virtual review. High risk patients (more than 7 per cent risk of cancer) should undergo appropriate cross-sectional imaging and/or ultrasound, FNA cytology (FNAC), or core biopsy. In order to aid appropriate triage of a radiology request, the head and neck cancer risk calculator score is included in all requests.

The FNA or core biopsy should be performed at the same time as the ultrasound scan, based on radiological suspicion. If the ultrasound images are concerning for malignancy, the patient will be sent for cross-sectional imaging during the same visit. Conversely, patients sent for cross-sectional imaging would ideally be scanned on days that dedicated head and neck radiologists perform ultrasound scans. Imaging is reviewed before the patient leaves the department so that the ultrasound scan or FNA can be undertaken if required.

If suspicion of malignancy remains following these investigations, the patient will be advised to attend the next joint rapid diagnostic out-patient clinic for examination and flexible nasal endoscopy. Here, patients with a visible primary lesion site in the upper aero-digestive tract should undergo a targeted biopsy using a transnasal oesophagoscope, or an ultrasound-guided (percutaneous) FNA or core biopsy. Patients with suspicious neck lumps will undergo ultrasound-guided FNA or core biopsy. If tissue diagnosis is not possible in the clinic, the patients will be discussed at a multidisciplinary team (MDT) meeting prior to biopsy, to consider primary excisional resection with or without neck dissection. This will also be a consideration for patients with an occult primary lesion, after positron emission tomography computed tomography (PET-CT) has been carried out.

Patients referred by the haematology-oncology department with ‘B symptoms’ and rapid enlargement of a neck lump should ideally undergo an ultrasound-guided core biopsy prior to clinical review. If core biopsy findings are inconclusive, a diagnostic lymph node excision should be performed, and the results discussed at the haematology-oncology MDT meeting, together with discussions of PET-CT findings.

There is differing guidance regarding the investigation of thyroid nodules between the British Society of Head and Neck Imaging^6^ and the British Association of Endocrine and Thyroid Surgeons.^7^ Our thyroid MDT follows the British Society of Head and Neck Imaging guidelines; these recommend postponing ultrasound scanning unless thyroid masses ‘rapidly expand over a few weeks, are associated with breathing or swallowing difficulty, or there are palpable metastatic lymph nodes’.^6^ In contrast, the British Association of Endocrine and Thyroid Surgeons do not recommend restricting ultrasound scanning, but advise limiting FNA, depending on the ultrasound ‘U’ classification and size of thyroid nodules. NHS England recommends proven thyroid cancer resection within one month and diagnostic hemi-thyroidectomy within three months.^8^

Our preliminary experience is that a definitive diagnosis of a thyroid nodule may be difficult to elicit over the phone. The head and neck cancer risk calculator, version 2, will universally put persistent neck lump patients into a high risk category, necessitating further investigation. One could argue that the risk calculator is not suitable for thyroid neoplasms, but unfortunately, at present, we cannot determine how to differentiate thyroid from non-thyroid lumps virtually, and most patients will therefore be advised to undergo ultrasound scanning with or without FNAC. The results will be discussed in the thyroid MDT meeting to determine the urgency of diagnostic or definitive surgery.

## Results

Our early results indicate the feasibility of our model. Of 48 patients referred via the two-week wait pathway, 15 were stratified as low risk (31 per cent). Twelve of the 15 patients (80 per cent) were discharged without imaging. Two patients’ findings warranted a further virtual review in eight weeks, and one patient requested imaging in the absence of clinical review.

Seventeen patients were stratified as intermediate risk (35 per cent). Eleven of these 17 patients (65 per cent) had imaging arranged prior to further virtual clinic follow up, and 4 of the 17 (24 per cent) were discharged without imaging. Two of the 17 patients (12 per cent) had further virtual clinic follow up without prior imaging.

Across all risk groups, a total of 16 patients (33 per cent) were discharged without imaging. In comparison, the introduction of a rapid diagnostic clinic at our institution in 2013 led to 27 per cent of patients being discharged after their first appointment.^9^

There were 16 high risk patients (33 per cent); 13 of these (81 per cent) had imaging arranged prior to further virtual review. One patient had imaging and clinical review, and a further patient had clinical review only. One high risk patient was discharged back to the general practitioner with a letter to the referrer, as they were not contactable and did not attend two appointments.

A collaborative remote MDT, consisting of a head and neck radiologist and a consultant ENT surgeon, re-triaged all pending imaging requests (organised pre-Covid-19). Ten new two-week wait referral imaging requests were vetted retrospectively using the head and neck cancer risk calculator, and re-stratified as low, intermediate or high risk. All the low risk patients had their magnetic resonance imaging (MRI) cancelled and were offered a delayed clinical review via telephone. The planned imaging was retained for one intermediate risk patient and was replaced with clinical review for another patient. The patient stratified as high risk had their imaging request upheld. All future requests will document the risk calculation (based on the head and neck cancer risk calculator, version 2) to help the vetting process.

- The coronavirus disease 2019 pandemic requires immediate modification to existing head and neck two-week wait pathways
- A robust triaging tool with risk stratification must be used to focus resources and highlight most at-risk patients
- Low risk patients should be referred back to primary care, with the safety net of advice and consideration of a primary care follow-up consultation
- Clinical encounters and aerosol generating procedures should be minimised to reduce the risk of virus transmission
- A combined diagnostic and therapeutic surgical approach should be undertaken where possible to reduce the surgical procedures required

## Discussion

Our pathway reduces the risk of Covid-19 transmission to patient and clinician, whilst maintaining rapid diagnosis and effective management of head and neck cancer. This is achieved through risk stratification of referrals and early investigations prior to clinical review. Patients in whom tissue cannot be obtained in the clinic should be discussed at the MDT meeting without histopathology, for MDT endorsement of a diagnostic excision biopsy and treatment strategy for that patient.

Implementing this pathway will reduce the future burden on tertiary services, by empowering primary care physicians to re-refer low risk patients. It utilises a nationally recognised statistical model of predicting the risk of head and neck cancer.^3^ The number of clinical encounters and aerosol generating procedures are reduced, decreasing the risk of spread of Covid-19. The specialist workforce is therefore kept healthy, allowing them to continue treating patients. Reducing the risk of exposing potential head and neck cancer patients to Covid-19 may give them a better chance of overall survival.

The risk of imaging missing a small mucosal lesion is low, but should not be overlooked. The sensitivity of MRI in detecting the primary lesion in a case series of 65 patients was 98 per cent.^10^ If symptoms are concerning in the presence of a normal scan, the clinician has the option of bringing the patient in for face-to-face review. Robust safety-netting, achieved by keeping moderate or high risk patients under surveillance in the head and neck clinic, and by asking general practitioners to follow up low risk patients, should mitigate the risk. In clinic letters, general practitioners are asked to re-refer if symptoms become progressive.

A future improvement for the pathway will be the utilisation of video consultation, which is currently being reviewed by our hospital trust. This will aid in the assessment of neck lump location, improving differentiation between thyroid lumps and cervical lymphadenopathy for example.

Risk-stratifying patients during the initial telephone consultation and the re-triage of existing requests has created an evidence-based strategy for performing, delaying or cancelling imaging. This ensures equitable decision-making, aids the radiology team in deciding which diagnostics to continue, and enhances communication with patients. During the pandemic, the requirement for imaging has been limited to emergency and oncology work. The use of the risk calculator has prevented an increase in burden on the radiology service, meaning there has always been the capacity to scan patients on the two-week wait pathway. Going forward, current social distancing rules must be taken into consideration, as they lead to reduced capacity in the imaging department.

Givi *et al*. (2020) advise postponing non-urgent head and neck consultations, and only examining patients if urgent.^11^ However, there are no detailed recommendations on adapting diagnostic pathways in the literature. We are all experiencing a paradigm shift in our management of head and neck cancer, and time will tell how we fared. It is of the utmost importance that we share our experiences, to optimise management of these patients whilst minimising the risk to healthcare professionals.

We have, at this unprecedented time, tried to uphold the two-week wait diagnostic pathway, despite the exceptional burden on our resources. The protocol is applicable across the UK and can be adapted to suit local practice. It avoids delays in diagnostics, with no increase in workload post-pandemic. If deemed successful, the protocol could be adapted after the pandemic to engage general practitioners in the investigation and management of low risk patients.
